# Supplementary material for: CryoEM reveals the stochastic nature of individual ATP binding events in a group II chaperonin
Source: Nat Commun. 2021 Aug 6;12:4754. doi: 10.1038/s41467-021-25099-0 (PMC8346469; doi:10.1038/s41467-021-25099-0)
Supplement: Supplementary file 1 — Supplementary information [file 41467_2021_25099_MOESM1_ESM.pdf]

# SUPPLEMENTARY INFORMATION

# Cryo-EM reveals the stochastic nature of individual ATP binding events in a group II chaperonin

Yanyan Zhao[1], Michael F. Schmid[2], Judith Frydman[1,3,4,5] and Wah Chiu[1,2,6]*

[1]Biophysics Graduate Program, Stanford University, Stanford, CA 94035.

[2]Division of CryoEM and Bioimaging, SSRL, SLAC National Accelerator Laboratory, Menlo Park, CA 94025.

[3]Department of Biology, Stanford University, Stanford, CA 94035.

[4]Department of Genetics, Stanford University, Stanford, CA 94035.

[5]CZ Biohub, San Francisco, CA.

[6]Department of Bioengineering, James Clark Center, Stanford University, Stanford, CA 94035.

*email: wahc@stanford.edu

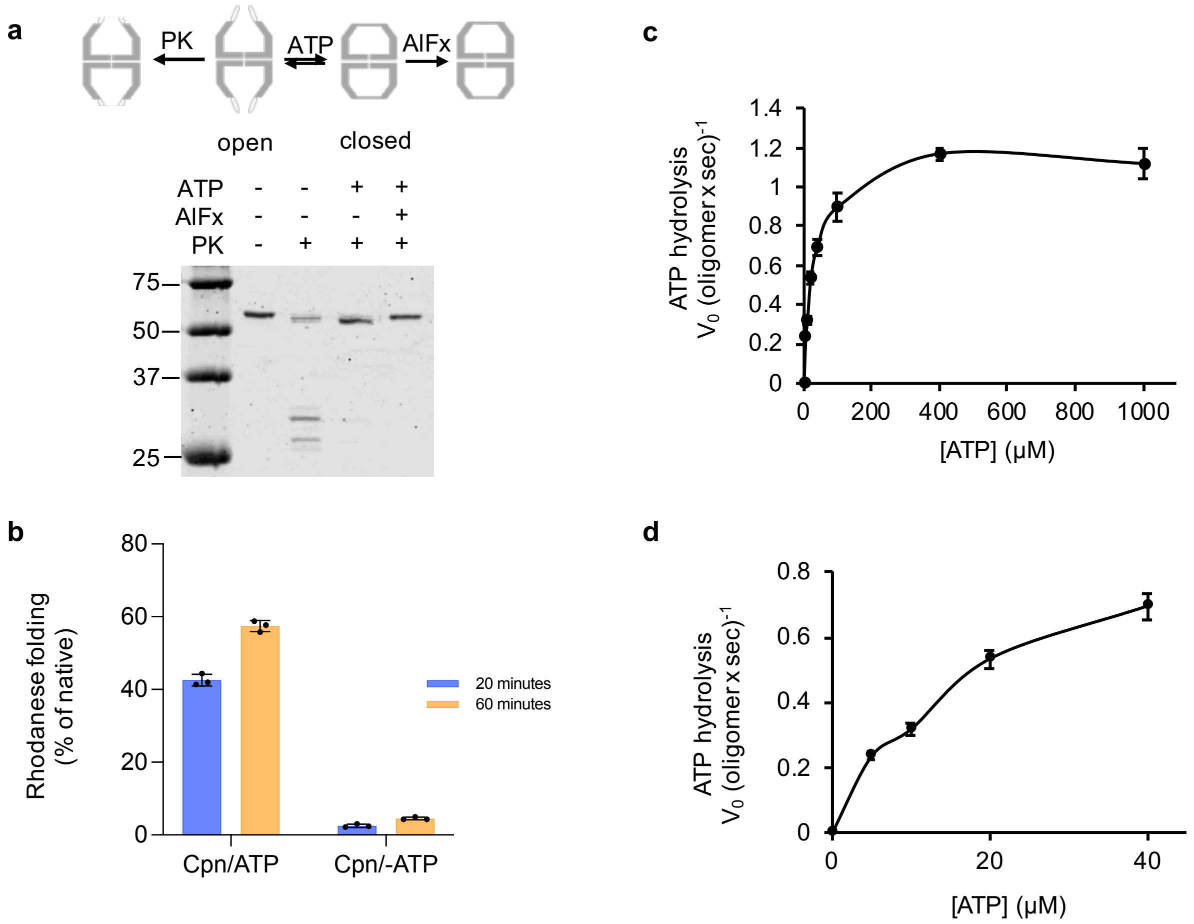

a
PK
ATP
AlFx
open
closed
ATP - - + +
AlFx - - - +
PK - + + +
75
50
37
25
b
Rhodanese folding (% of native)
20 minutes
60 minutes
Cpn/ATP
Cpn/-ATP
c
ATP hydrolysis $V_0$ (oligomer x sec)$^{-1}$
[ATP] (μM)
d
ATP hydrolysis $V_0$ (oligomer x sec)$^{-1}$
[ATP] (μM)

**Supplementary Figure 1**. **The MmCpn preparation used in this study is fully active and functional.** (a) Proteinase K (PK) digestion of incubations under three conditions. PK assay was repeated twice independently with similar result. Lane 1 is a negative control. Lane 2 shows that MmCpn is digested by proteinase K in the open state in the absence of ATP. Lane 3 shows the PK digestion of MmCpn during ATP cycling. A small portion of MmCpn that exists transiently in the open state during ATP cycling can be digested by proteinase K, while the majority in the closed form is protected from proteinase K digestion. Lane 4 shows no observable proteinase K digestion, demonstrating that all particles in our preparation are functional and reach closed state upon ATP binding and hydrolysis in the presence of AlFx. In the closed state, AlFx replaces ATP hydrolysis product Pi to interact with ADP and side chains and thus locks MmCpn in the closed state. The schematic is adaptated from figure by Kevin Dalton *et al*[1] licensed under CC BY 4.0. (b) Rhodanese folding by MmCpn at 20 mins and 60 mins intervals. Data is normalized against the activity of 1 μM native rhodanese. Data are the mean ± S.D. of three independent replicates. MmCpn can efficiently fold rhodanese in an ATP dependent manner, which is indicative of a fully active preparation. (c) Steady state ATPase measurements at a range of ATP concentrations showing expected allosteric regulation. Rates shown were measured using an enzyme-coupled assay for ADP generated calculated by monitoring NADH oxidation at 340 nm. Data are the mean ± S.D. of three independent replicates. (d) Enlarged view of nucleotide hydrolysis rates of MmCpn at low ATP concentration range from c.

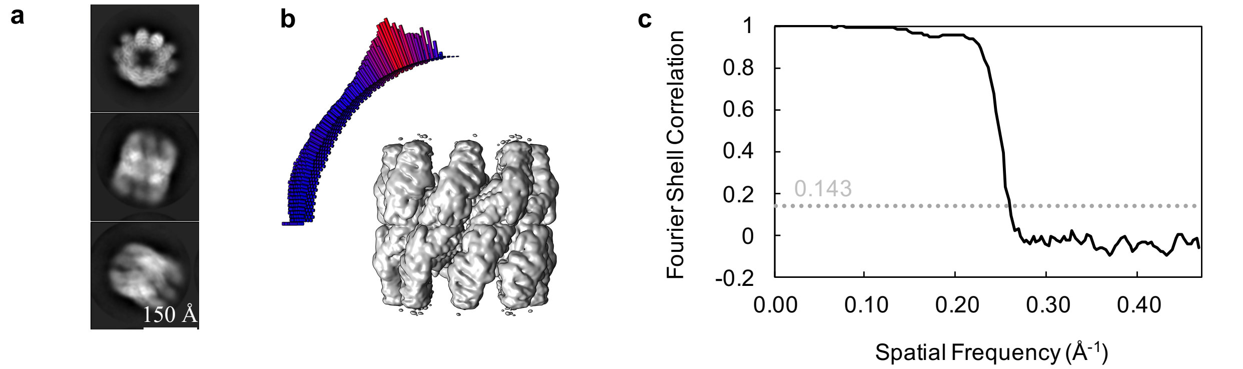


**Supplementary Figure 2. Cryo-EM reconstruction with D8 symmetry.** (a) Reference-free 2D class averages. (b) Euler angle distribution of all particles used in D8 symmetry-imposed map refinement. (c) FSC curves of masked density map showing resolution of 3.9 Å according to the gold standard FSC (0.143 criterion).

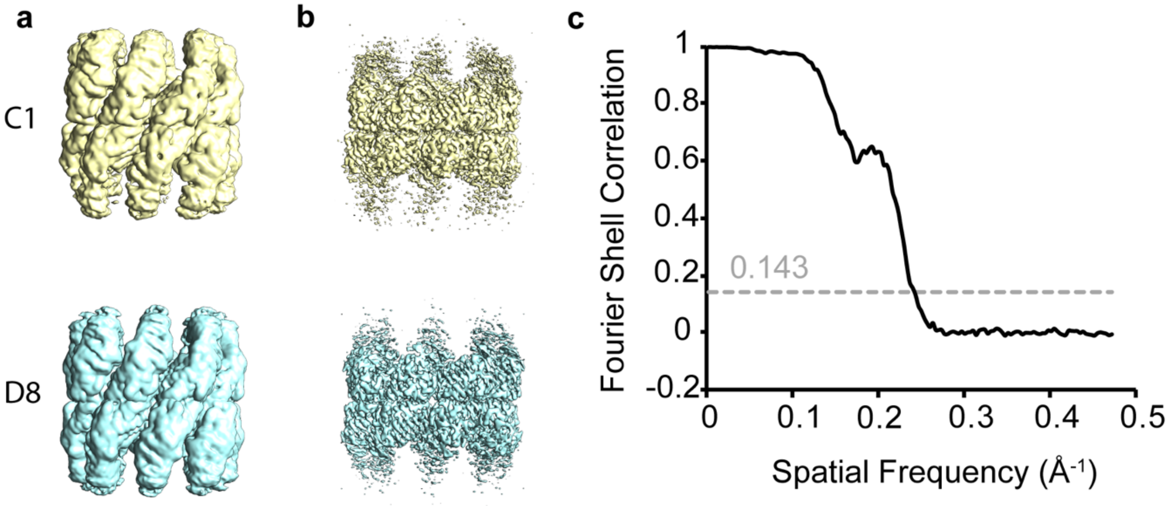


**Supplementary Figure 3. D8 symmetry imposed reconstruction vs no symmetry imposed reconstruction.** (a) B-factor sharpened maps of C1 (yellow) and D8 symmetry-imposed (cyan) reconstructions. (b) Segmented cryo-EM map for a single subunit from C1 reconstruction (yellow) and D8 symmetry-imposed (cyan) reconstructions in a. The equatorial domains of both maps show the alpha helices features whereas the apical domains remain poorly resolved due to conformational heterogeneity within and across particles. (c) FSC curve of masked C1 density map showing resolution of 4.3 Å according to the gold standard FSC (0.143 criterion).

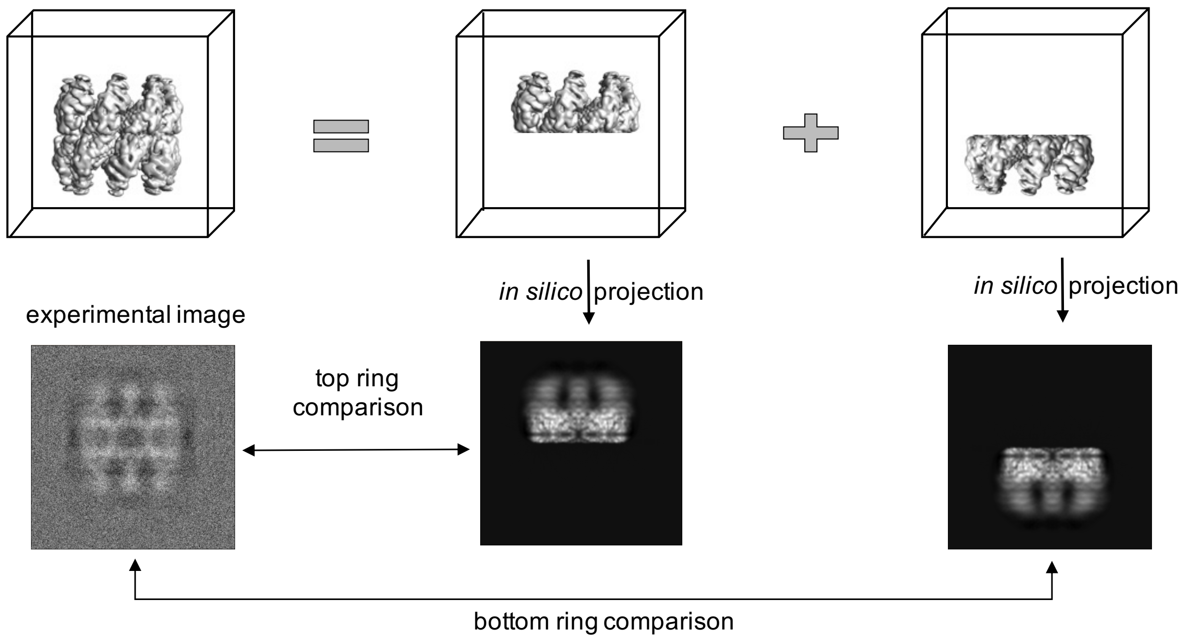


**Supplementary Figure 4. Masked classification of two rings in each particle image.** In this approach, the experimental particle is compared with reference projections of each ring.

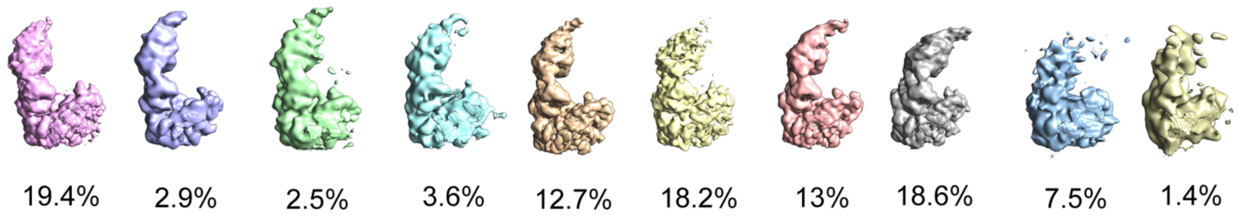


**Supplementary Figure 5. Subunit-focused classification into ten classes of individual subunits.** The subunit classes are shown in lower threshold than in Figure 2b in order to show the presence of apical domain density.

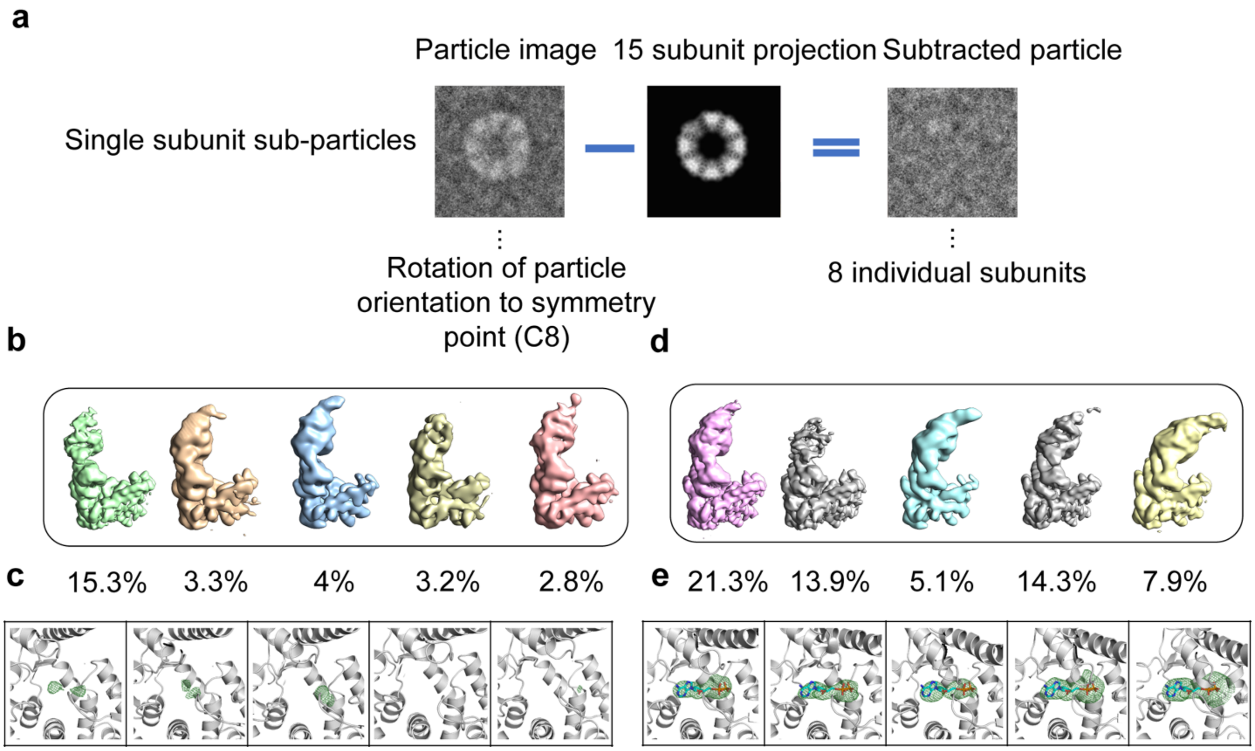


**Supplementary Figure 6. Subunit focused classification with signal subtraction.** (a) Signal subtraction of the projection including 15 subunits from original particles results in single subunit subparticles. (b) Subunit-focused classification of subunits from class 1 to class 4 with partial signal subtraction. (c) Difference map analysis of the nucleotide occupancy in subunit conformations from b. (d) Subunit-focused classification of subunits from class 5 to class 8 with partial signal subtraction. (e) Difference map analysis of the nucleotide occupancy in subunit conformations from d. The difference maps are displayed with +4σ.

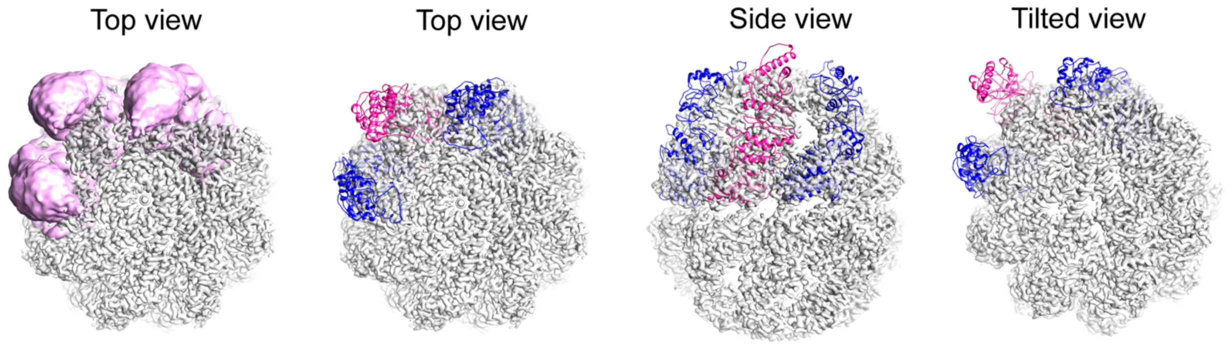


**Supplementary Figure 7. The open subunit flanked by two closed subunits.** From left to right are reconstructed map of triplet (pink) and model (open subunit in magenta, and closed subunits in blue) superimposed on a full closed map (gray).

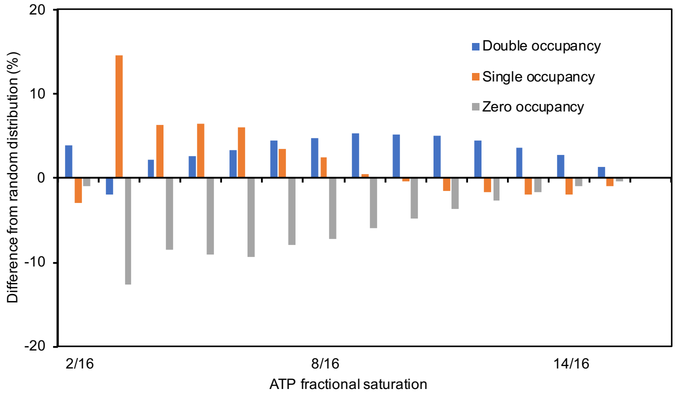


**Supplementary Figure 8. The difference of the nucleotide occupancy in inter-ring double subunits between Figure 5b and random distribution.** It is calculated by subtracting the prediction based on random distribution at each saturation from the observation in Figure 5b.

**Supplementary Table 1. Statistics for cryo-EM data collection and processing and model validation.**

| | |
|---|---|
| **Data collection** | |
| Number of grids used | 1 |
| Grid type | Quantifoil holey carbon grid (R2/1) |
| Microscope/detector | Titan Krios/Gatan K2 |
| Voltage | 300 kV |
| Magnification | 130, 000x |
| Recording mode | Counting mode |
| Dose rate at the detector | 5 $e^{-}$/sec/$Å^2$ |
| Defocus | -0.8 to -2.0 µm |
| Pixel size | 1.08 Å/pixel |
| Total specimen dose | 50 $e^{-}$/$Å^2$ |
| Frame rate | 5 frames per second |
| Number of frames/movie | 50 |
| Total exposure time | 10 sec |
| Number of micrographs | 6, 500 |
| **Refinement** | |
| Total particles picked | 971, 741 |
| Particles used for final map | 165, 212 |
| Map resolution (FSC 0.143) | 3.9 Å |
| Symmetry | D8 |
| Local resolution (Monores) | 3.5 Å - >10 Å |
| **Validation** | |
| MolProbity score | 1.83 |
| Clashscore | 3.43 |
| Poor rotamers (%) | 1.63 |
| Ramachandran plot | |
| Favored (%) | 90.50 |
| Allowed (%) | 8.71 |
| Outliers (%) | 0.79 |

**Supplementary Table 2. Validation of models refined with phenix against subunit density maps (Figure 2b) from focused classification without partial signal subtraction.**

| | Class 1 | Class 2 | Class 3 | Class 4 | Class 5 | Class 6 | Class 7 | Class 8 |
|---|---|---|---|---|---|---|---|---|
| Resolution (Å) | 4.0 | 6.3 | 6.4 | 6.3 | 4.0 | 4.0 | 4.4 | 4.1 |
| MolProbity score | 1.61 | 1.63 | 1.61 | 1.69 | 1.58 | 1.61 | 1.59 | 1.59 |
| Clashscore | 2.46 | 3.11 | 2.72 | 2.85 | 2.20 | 2.98 | 2.59 | 2.33 |
| Poor rotamers (%) | 0.00 | 0.25 | 0.00 | 0.25 | 0.25 | 0.00 | 0.00 | 0.00 |
| Map/model correlation | 0.80 | 0.69 | 0.64 | 0.58 | 0.71 | 0.77 | 0.62 | 0.71 |
| **Ramachandran plot** | | | | | | | | |
| Favored (%) | 88.87 | 90.66 | 90.06 | 87.08 | 88.67 | 90.85 | 90.26 | 89.07 |
| Allowed (%) | 9.74 | 8.95 | 9.34 | 10.93 | 10.54 | 8.35 | 9.15 | 10.34 |
| Outliers (%) | 1.39 | 0.40 | 0.60 | 1.99 | 0.80 | 0.80 | 0.60 | 0.60 |

**Supplementary Table 3. Validation of models refined with phenix against subunit density maps (shown in Supplementary Figure 5b) from focused classification with partial signal subtraction.**

| | Class 1 | Class 2 | Class 3 | Class 4 | Class 5 |
|---|---|---|---|---|---|
| Resolution (Å) | 4.3 | 6.9 | 6.7 | 7.0 | 7.0 |
| MolProbity score | 1.71 | 1.64 | 1.59 | 1.75 | 1.76 |
| Clashscore | 2.85 | 2.59 | 2.46 | 2.98 | 2.72 |
| Poor rotamers (%) | 0.25 | 0.25 | 0.00 | 0.00 | 0.00 |
| **Ramachandran plot** | | | | | |
| Favored (%) | 86.48 | 88.27 | 89.46 | 85.09 | 82.50 |
| Allowed (%) | 12.33 | 10.74 | 9.94 | 13.72 | 16.10 |
| Outliers (%) | 1.19 | 0.99 | 0.60 | 1.19 | 1.39 |

**Supplementary Table 4. Validation of models refined with phenix against subunit density maps (shown in Supplementary Figure 5d) from focused classification with partial signal subtraction.**

| | Class 1 | Class 2 | Class 3 | Class 4 | Class 5 |
|---|---|---|---|---|---|
| Resolution (Å) | 4.0 | 4.4 | 6.9 | 4.4 | 5.5 |
| MolProbity score | 1.61 | 1.55 | 1.64 | 1.62 | 1.99 |
| Clashscore | 2.46 | 1.94 | 2.46 | 2.33 | 4.14 |
| Poor rotamers (%) | 0.00 | 0.00 | 0.00 | 0.00 | 1.47 |
| **Ramachandran plot** | | | | | |
| Favored (%) | 88.87 | 88.47 | 87.48 | 87.48 | 84.49 |
| Allowed (%) | 10.34 | 10.74 | 12.13 | 11.93 | 14.51 |
| Outliers (%) | 0.80 | 0.80 | 0.40 | 0.60 | 0.99 |

**Supplementary Table 5. Validation of models refined with phenix against intra-ring triple subunit density map and inter-ring double subunit maps.**

| | Intra-ring triple subunits | Inter-ring closed/closed subunits | Inter-ring closed/open subunits |
|---|---|---|---|
| Resolution (Å) | 6.2 | 6.0 | 4.6 |
| MolProbity score | 1.69 | 1.88 | 1.65 |
| Clashscore | 3.02 | 5.11 | 3.18 |
| Poor rotamers (%) | 0.08 | 0.98 | 0.49 |
| Map/model correlation | 0.85 | 0.82 | 0.55 |
| **Ramachandran plot** | | | |
| Favored (%) | 88.14 | 87.77 | 90.24 |
| Allowed (%) | 10.40 | 11.43 | 8.86 |
| Outliers (%) | 1.46 | 0.80 | 0.90 |

## Supplementary References


1. Dalton, K., Lopez, T., Pande, V. & Frydman, J. REP-X: An Evolution-guided Strategy for the Rational Design of Cysteine-less Protein Variants. *Sci. Rep.* (2020). doi:10.1038/s41598-020-58794-x
