# Supplementary material for: CryoEM reveals the stochastic nature of individual ATP binding events in a group II chaperonin
Source: Nat Commun. 2021 Aug 6;12:4754. doi: 10.1038/s41467-021-25099-0 (PMC8346469; doi:10.1038/s41467-021-25099-0)
Supplement: Supplementary file 3 — Description of Additional Supplementary Files [file 41467_2021_25099_MOESM3_ESM.pdf]

Description of Additional Supplementary Files

Supplementary Movie 1. Example of the conformational heterogeneity in chaperonin MmCpn oligomer. Colors of subunits in each oligomer are consistent with colors for the subunit density maps. Two examples are compared side by side to show intra-ring adjacent subunits can adopt either the same conformation or different conformations.
