# Supplementary material for: CryoEM reveals the stochastic nature of individual ATP binding events in a group II chaperonin
Source: Nat Commun. 2021 Aug 6;12:4754. doi: 10.1038/s41467-021-25099-0 (PMC8346469; doi:10.1038/s41467-021-25099-0)
Supplement: Supplementary file 5 — Reporting summary [file 41467_2021_25099_MOESM5_ESM.pdf]

nature portfolio

Corresponding author(s): Wah Chiu

Last updated by author(s): Jul 11, 2021

Reporting Summary

Nature Portfolio wishes to improve the reproducibility of the work that we publish. This form provides structure for consistency and transparency in reporting. For further information on Nature Portfolio policies, see our Editorial Policies and the Editorial Policy Checklist.

Statistics

For all statistical analyses, confirm that the following items are present in the figure legend, table legend, main text, or Methods section.

n/a	Confirmed	
☐	☒	The exact sample size (n) for each experimental group/condition, given as a discrete number and unit of measurement
☒	☐	A statement on whether measurements were taken from distinct samples or whether the same sample was measured repeatedly
☒	☐	The statistical test(s) used AND whether they are one- or two-sided<br>*Only common tests should be described solely by name; describe more complex techniques in the Methods section.*
☒	☐	A description of all covariates tested
☒	☐	A description of any assumptions or corrections, such as tests of normality and adjustment for multiple comparisons
☐	☒	A full description of the statistical parameters including central tendency (e.g. means) or other basic estimates (e.g. regression coefficient) AND variation (e.g. standard deviation) or associated estimates of uncertainty (e.g. confidence intervals)
☒	☐	For null hypothesis testing, the test statistic (e.g. F, t, r) with confidence intervals, effect sizes, degrees of freedom and P value noted<br>*Give P values as exact values whenever suitable.*
☒	☐	For Bayesian analysis, information on the choice of priors and Markov chain Monte Carlo settings
☒	☐	For hierarchical and complex designs, identification of the appropriate level for tests and full reporting of outcomes
☒	☐	Estimates of effect sizes (e.g. Cohen's d, Pearson's r), indicating how they were calculated

Our web collection on statistics for biologists contains articles on many of the points above.

Software and code

Policy information about availability of computer code

Data collection	We use Thermo Fisher EPU v1.10 to collect cryo-EM images of sample in an automated manner on a single grid.
Data analysis	We use MotionCor2 v1.0.0, Gctf v1.0.6, RELION from MRC v3.0.1 for single particle analysis, and Chimera v1.14, Pymol v2.3.2 for display, and Phenix v1.18.1 and Coot V0.9.5,ISOLDE v1.1.0 for model refinement.

For manuscripts utilizing custom algorithms or software that are central to the research but not yet described in published literature, software must be made available to editors and reviewers. We strongly encourage code deposition in a community repository (e.g. GitHub). See the Nature Portfolio guidelines for submitting code & software for further information.

Data

Policy information about availability of data

All manuscripts must include a data availability statement. This statement should provide the following information, where applicable:

- Accession codes, unique identifiers, or web links for publicly available datasets
- A description of any restrictions on data availability
- For clinical datasets or third party data, please ensure that the statement adheres to our policy

The 3D cryoEM density maps are deposited in the Electron Microscopy Data Bank (accession no. EMD-24324, EMD-24325, EMD-24326, EMD-24327, EMD-24328, EMD-24329, EMD-24330, EMD-24331, EMD-24363). The atomic coordinates are deposited in the Protein Data Bank, www.pdb.org (PDB ID code 7R9H, 7R9E, 7R9I, 7R9J, 7R9O, 7R9M, 7R9U, 7R9K, 7RAK). Source image data are deposited to EMPIAR.

Field-specific reporting

Please select the one below that is the best fit for your research. If you are not sure, read the appropriate sections before making your selection.

- [x] Life sciences
- [ ] Behavioural & social sciences
- [ ] Ecological, evolutionary & environmental sciences

For a reference copy of the document with all sections, see nature.com/documents/nr-reporting-summary-flat.pdf

Life sciences study design

All studies must disclose on these points even when the disclosure is negative.

Sample size	The number of particles used to determine the ATP occupancy and distribution is decided based on the resolvability of the reconstructed map.
Data exclusions	To avoid the over-representation of the images with two major preferred orientations in the final reconstructions, it is customary to equalize the contributions of different particles' orientations by reducing the number of included particles with these orientations for the final reconstruction.
Replication	The classification were performed twice independently with two different strategy. Subunits are first classified by focused classification without partial signal subtraction method, and the classified results are then confirmed by focused classification with partial signal subtraction method.
Randomization	In 3D auto-refinment, the total dataset of particles is randomly split into two half sets and for each set, a 3D reconstruction is calculated and two maps are compared in Fourier space.
Blinding	The investigator is blinded during the data collection in terms of the ATP occupancy in each chaperonin complex. None or minimal amount of user intervention is involved in auto-refinement and 3D classification with software RELION, i.e.the investigator is blinded to group allocation (3D classification) in data analysis.

Reporting for specific materials, systems and methods

We require information from authors about some types of materials, experimental systems and methods used in many studies. Here, indicate whether each material, system or method listed is relevant to your study. If you are not sure if a list item applies to your research, read the appropriate section before selecting a response.

Materials & experimental systems

n/a	Involved in the study	
[x]	[ ]	Antibodies
[x]	[ ]	Eukaryotic cell lines
[x]	[ ]	Palaeontology and archaeology
[x]	[ ]	Animals and other organisms
[x]	[ ]	Human research participants
[x]	[ ]	Clinical data
[x]	[ ]	Dual use research of concern

Methods

n/a	Involved in the study	
[x]	[ ]	ChIP-seq
[x]	[ ]	Flow cytometry
[x]	[ ]	MRI-based neuroimaging
